# Inhibition of Fibrinolysis by Streptococcal Phage Lysin_SM1_

**DOI:** 10.1128/mBio.00746-21

**Published:** 2021-06-22

**Authors:** Hyun Jung Ji, Yong Zhi, Ji Hee Lee, Ki Bum Ahn, Ho Seong Seo, Paul M. Sullam

**Affiliations:** a Research Division for Radiation Science, Korea Atomic Energy Research Institute, Jeongeup, Republic of Korea; b Department of Radiation Science, University of Science and Technology, Daejeon, Republic of Korea; c Department of Oral Microbiology and Immunology, DRI, and BK21 Plus Program, School of Dentistry, Seoul National University, Seoul, Republic of Korea; d Department of Medicine, Veterans Affairs Medical Center and University of California, San Francisco, California, USA; University of Washington School of Medicine, Seattle Children’s Research Institute; Harvard Medical School

**Keywords:** *Streptococcus mitis*, fibrinogen, fibrinolysis, infective endocarditis, plasminogen, thromboelastography

## Abstract

Expression of bacteriophage lysin_SM1_ by Streptococcus oralis strain SF100 is thought to be important for the pathogenesis of infective endocarditis, due to its ability to mediate bacterial binding to fibrinogen. To better define the lysin_SM1_ binding site on fibrinogen Aα, and to investigate the impact of binding on fibrinolysis, we examined the interaction of lysin_SM1_ with a series of recombinant fibrinogen Aα variants. These studies revealed that lysin_SM1_ binds the C-terminal region of fibrinogen Aα spanned by amino acid residues 534 to 610, with an affinity of equilibrium dissociation constant (*K_D_*) of 3.23 × 10^−5^ M. This binding site overlaps the known binding site for plasminogen, an inactive precursor of plasmin, which is a key protease responsible for degrading fibrin polymers. When tested *in vitro*, lysin_SM1_ competitively inhibited plasminogen binding to the αC region of fibrinogen Aα. It also inhibited plasminogen-mediated fibrinolysis, as measured by thromboelastography (TEG). These results indicate that lysin_SM1_ is a bi-functional virulence factor for streptococci, serving as both an adhesin and a plasminogen inhibitor. Thus, lysin_SM1_ may facilitate the attachment of bacteria to fibrinogen on the surface of damaged cardiac valves and may also inhibit plasminogen-mediated lysis of infected thrombi (vegetations) on valve surfaces.

## INTRODUCTION

Infective endocarditis (IE) is a life-threatening disease of cardiac valves that can lead to complications such as congestive heart failure and stroke and has an overall mortality rate of 30% (1, 2). The pathogenesis of IE is a complex process, involving numerous host-pathogen interactions (1, 3). A key interaction for disease establishment and progression is the binding of microbes to human components, including platelets, fibrinogen, fibrin, and fibronectin on damaged endocardium (3–11).

Streptococcus oralis *and* Streptococcus mitis are closely related members of the oral microbiome. These two species cannot be reliably distinguished by conventional microbiologic testing or 16S ribosome genotyping. Instead, accurate identification to the species level of these organisms requires more advanced methods, such as genome-wide association studies (12), and for this reason, clinical isolates are sometimes identified as “Streptococcus mitis/*oralis*” (13–17). These organisms are a leading cause of IE, with mortality ranging from 6% to 30% (18, 19). Despite the increasing importance of endocarditis due to S. mitis/*oralis*, especially in view of the high prevalence of multidrug resistance among these strains, relatively little is known about the virulence determinants of S. mitis/*oralis*. Our previous studies have identified several surface adhesins of S. oralis strain SF100 (formerly identified as S. mitis), such as PblA, PblB, and lysin_SM1_, that mediate binding to human platelets and enhance virulence in animal models of IE (20–23). Lysin_SM1_ is encoded by a lysogenic bacteriophage (SM1) and has been shown to have at least two pathogenetic functions. First, lysin is essential for the export of the phage-encoded adhesins, PblA and PblB. In addition, extracellular lysin can bind phosphocholine residues on the bacterial cell wall, where it can mediate bacterial binding to fibrinogen (21). Deletion of the lysin_SM1_ gene in SF100 resulted in significantly lower binding of the organism to fibrinogen and platelets *in vitro* and delayed the onset of platelet aggregation by this strain (20).

**FIG 1:**
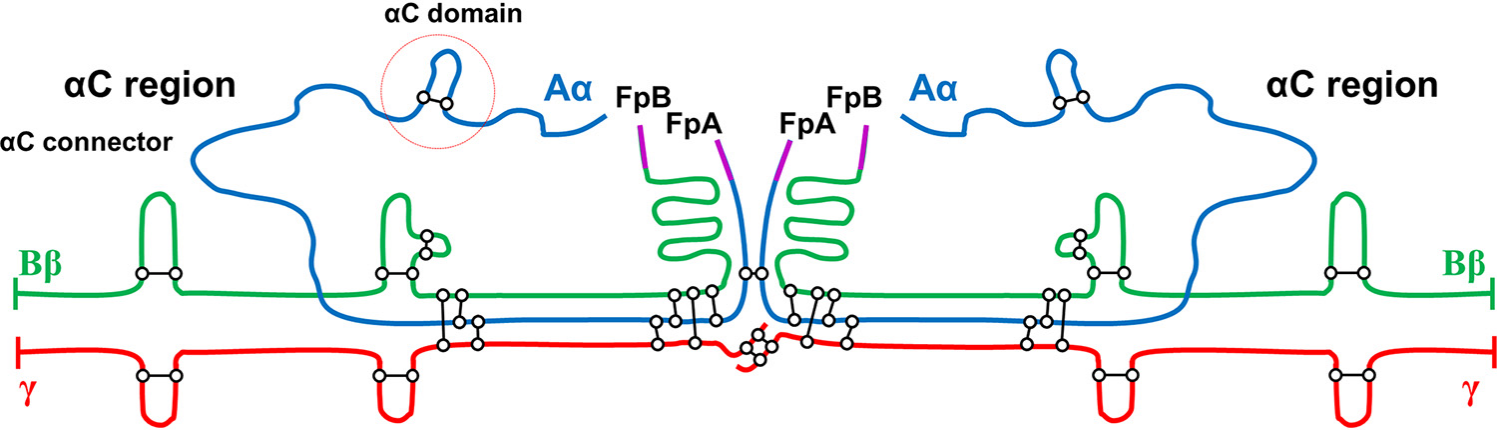
Schematic diagram of the intact fibrinogen dimer. The individual chains, Aα, Bβ, and γ, are blue, green, and red, respectively. Fibrinopeptides A and B (FpA and FpB) are magenta, and the disulfide bonds are shown by black bars. αC domains are consistent with the αC-domain and the flexible αC-connectors.

Fibrinogen is a 340-kDa glycoprotein comprising three pairs of distinct polypeptide chains (Aα, Bβ, and γ; Fig. 1) that are linked by 29 disulfide bridges (24, 25). It can be polymerized by the hydrolytic catalysis of its terminal ends by thrombin, resulting in fibrin clots or thrombi. Fibrin polymers can be degraded by a proteolytic process known as fibrinolysis, which is tightly controlled by a series of cofactors, inhibitors, and receptors (26–28). The high-affinity binding of plasminogen, a serine protease, to the distal portion of each αC region of fibrinogen Aα chains is the first step of fibrinolysis. Bound plasminogen is then activated to plasmin by cleavage at AA561 by tissue-type plasminogen activator (t-PA), thereby triggering fibrinolysis (29–31).

With a view toward better understanding how lysin_SM1_ interacts with fibrinogen, we identified the specific binding site for lysin_SM1_ within the fibrinogen Aα chain and investigated the effect of this interaction on clotting and fibrinolysis. Our studies indicate that a specific interaction of the binding domain in fibrinogen Aα chain overlaps a region bound by plasminogen. Moreover, binding of this region by lysin_SM1_ inhibits plasmin-mediated fibrinolysis.

## RESULTS

### Lysin_SM1_ binding to the αC region of fibrinogen Aα.

**FIG 2:**
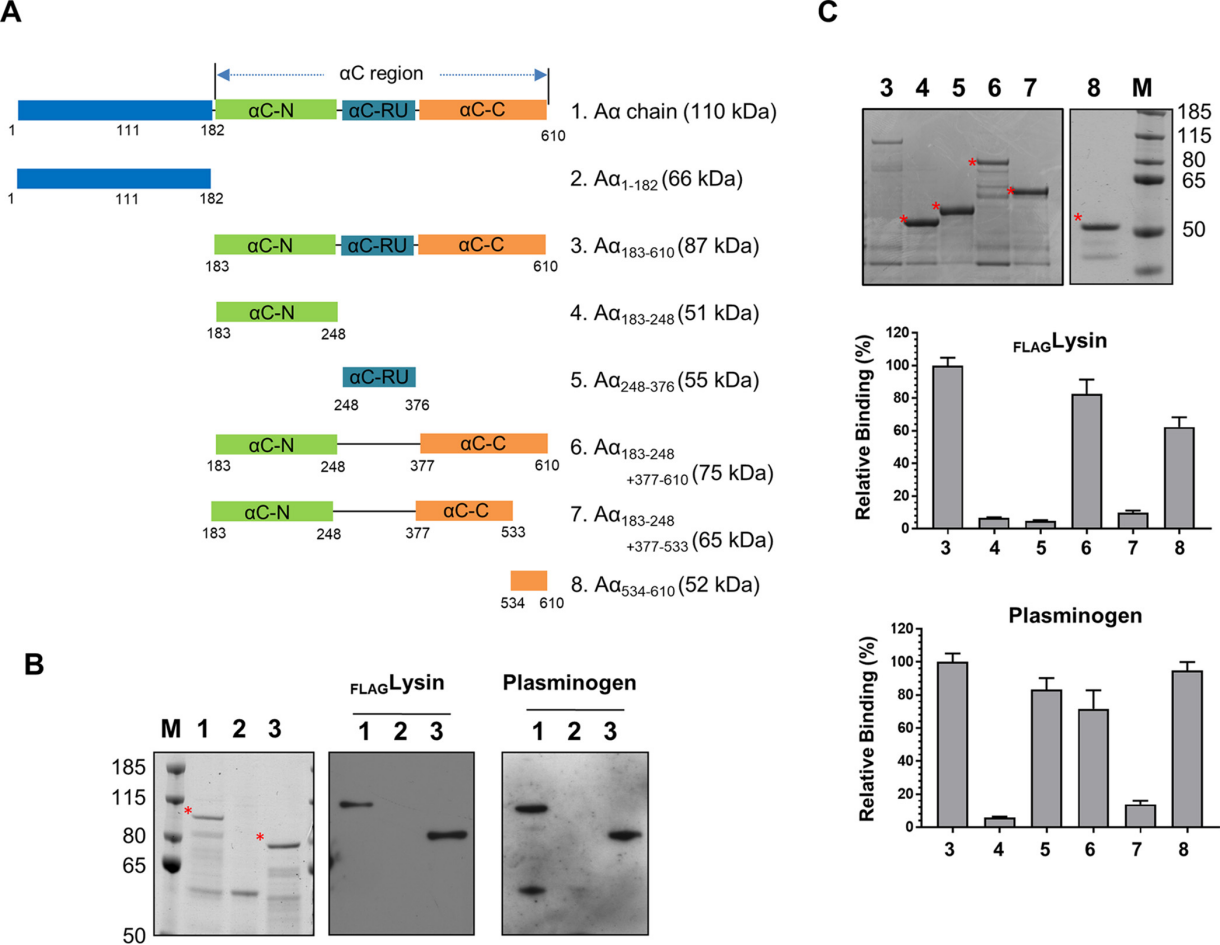
Identification of lysin_SM1_ and plasminogen binding regions on the fibrinogen Aα chain. (A) Schematic diagram of the fibrinogen Aα chain and its recombinant variants. All variants were expressed as MalE fusion proteins. (B) Binding of lysin_102-198_ (10 μg) or plasminogen (10 μg) to human fibrinogen Aα and Aα variants 2 and 3; analysis by far Western blotting. (C) Binding of lysin_102-198_ or plasminogen to immobilized recombinant fibrinogen Aα variants 3 to 8 (0.1 μM). Recombinant fibrinogen truncates were separated by SDS-PAGE and stained with Coomassie blue (top). * indicates the expected molecular sizes of recombinant variants. The binding region was identified by measuring lysin_102-198_ (1 μM; middle) or plasminogen (1 μM; bottom) binding to immobilized fibrinogen truncates. Bound proteins were detected with anti-FLAG or anti-plasminogen monoclonal antibodies.

We previously showed that recombinant lysin_SM1_ encoded by bacteriophage SM1 binds to the Aα chain of human fibrinogen and that this interaction enhances the attachment of S. oralis SF100 to human platelets (21). The domain of lysin that bound to the Aα chain was contained within the region spanned by amino acid residues 102 to 198 (97 amino acids [AA]) (20). To identify the regions within fibrinogen Aα that bound lysin_SM1_, we expressed and purified recombinant forms of the whole Aα (610 aa; variant 1), N-terminal region (AA1-182; variant 2) and C-terminal region (αC region; AA183-610; variant 3) and examined their binding by lysin_102-198_ by far-Western blotting (Fig. 2A and B). Lysin_102-198_ (10 μg) bound to variant 1 and 3, but not variant 2, indicating that the lysin_SM1_ binding domain was located on the αC region of Aα chain. To identify the specific binding regions within this domain, several soluble truncated forms of the region fused to MalE were isolated and tested for lysin_102-198_ binding (Fig. 2C). Recombinant lysin_102-198_ bound to the variants containing the region spanned by amino acid residues 534 to 610 (variant 8), the C-terminal end of fibrinogen Aα chain.

To better define the plasminogen binding sites on fibrinogen, we examined the binding of recombinant human plasminogen with the above-described fibrinogen Aα chain subdomains, as measured by far-Western blotting. As expected, plasminogen (10 μg) bound the αC region (variants 1 and 3; Fig. 2B). Plasminogen also interacted with an ∼60-kDa protein (Fig. 2B, lane 1), which was identified as a fragment of variant 1 by liquid chromatography-tandem mass spectroscopy (LC-MS/MS) (data not shown). In addition, plasminogen bound the variants containing the region spanned by amino acid residues 534 to 610 (variant 8) and 248 to 376 (variant 5; αC-RU) (Fig. 2C), indicating that it has two separate binding sites on the Aα chain (AA183-376 and AA534-610).

We next used overlapping 19-amino acid peptides fused with maltose binding protein (MBP) to localize the lysin binding segment between residues 534 and 610 of Fg Aα (Fig. S1). Lysin_SM1_ was shown to bind variants 8, 9, 10, and 12, but not variant 11 or 13, indicating that the binding peptide of the fibrinogen Aα chain is localized to AA572-590 (AGSEADHEGTHSTKRGHAK). To further demonstrate that lysin_SM1_ binding is specific, we made targeted point mutations within the lysin_SM1_ binding region (AA102-198) and assessed binding to the Fg Aα chain (Fig. S2). Of the four substitutions tested individually, both H111A and D188A were markedly reduced in binding to MalE:Aα_534-610_. These findings indicate that lysin_SM1_ binding to Fg Aα is specific and that these are key residues for this interaction.

10.1128/mBio.00746-21.1 FIG S1 Identification of the lysin_SM1_ binding region on the fibrinogen Aα chain. (A) Schematic diagram of recombinant variants of fibrinogen Aα_534-610_. All peptide variants were expressed as MAlE fusion proteins. (B) Binding of _FLAG_Lysin_102-198_ to Aα variants 8 to 13; analysis by far-Western blotting. Recombinant fibrinogen truncates (0.1 μM) were separated by SDS-PAGE and stained with Coomassie blue (top) or transferred to nitrocellulose and probed with FLAG-tagged lysin_102-198_ (1 μM; bottom). Bound proteins were detected with anti-FLAG (1:4,000 dilution). * indicates the expected molecular sizes of recombinant variants. Download FIG S1, TIF file, 0.9 MB. Copyright © 2021 Ji et al. 2021 Ji et al. https://creativecommons.org/licenses/by/4.0/ This content is distributed under the terms of the Creative Commons Attribution 4.0 International license.

10.1128/mBio.00746-21.2 FIG S2 Effects of point mutations on the binding of lysin_SM1_ to the fibrinogen Aα chain. Binding of _FLAG_lysin_SM1_ and its point mutants to immobilized fibrinogen Aα_534-610_ as measured by ELISA. (A to D) WT versus (A) S37C, (B) H111A, (C) S183A, and (D) D188A. (E) Stability and purity of recombinant lysin variants; 10 μg of recombinant _FLAG_lysin_SM1_ (WT) and the above variants were separated by SDS-PAGE and stained with Coomassie blue. Download FIG S2, TIF file, 0.6 MB. Copyright © 2021 Ji et al. 2021 Ji et al. https://creativecommons.org/licenses/by/4.0/ This content is distributed under the terms of the Creative Commons Attribution 4.0 International license.

**FIG 3:**
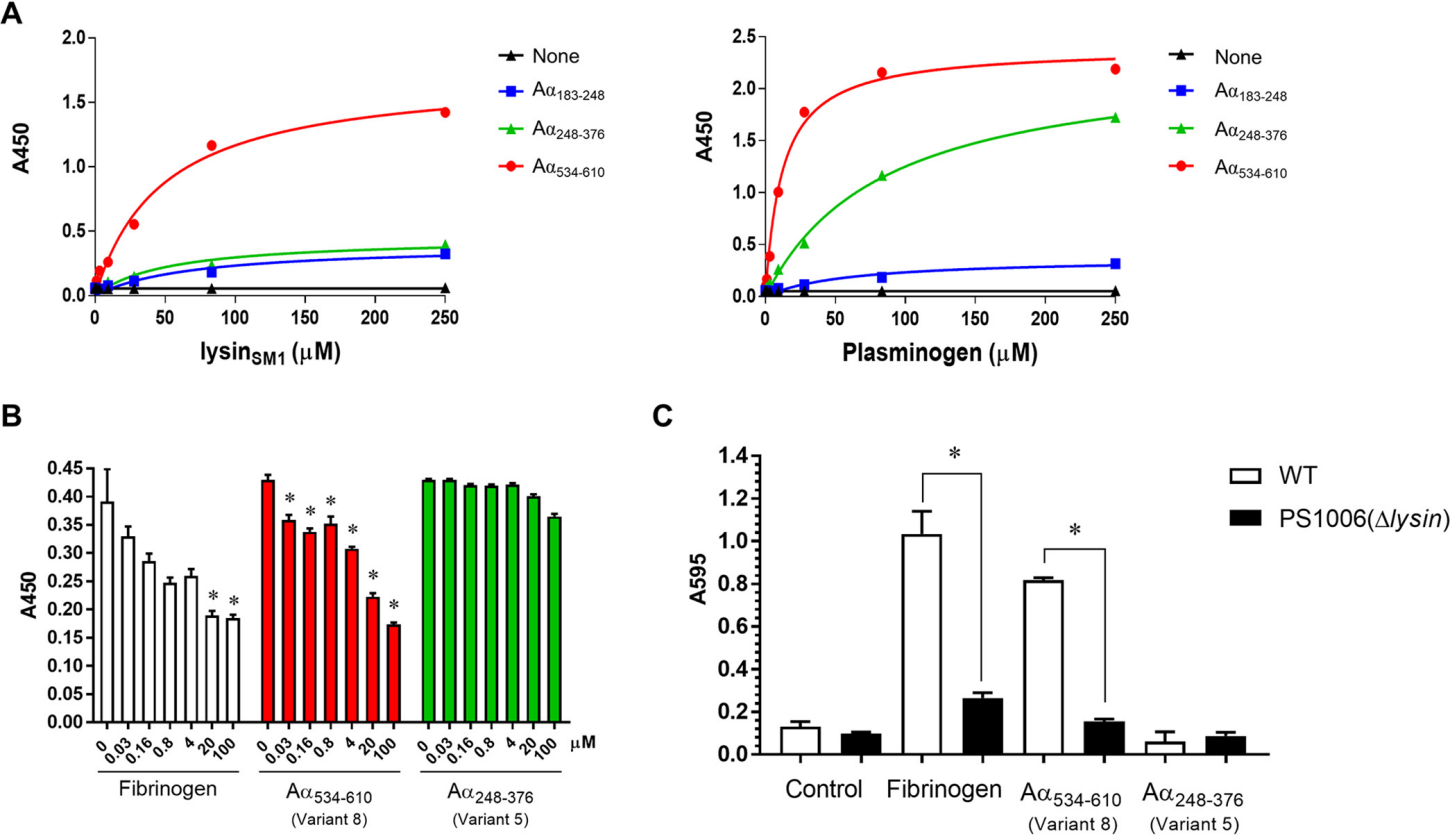
Quantitative analysis of fibrinogen binding by lysin_SM1_, plasminogen, and streptococci. (A) Binding of _FLAG_lysin_102-198_ (left) and plasminogen (right) to immobilized fibrinogen Aα variants 4, 5, or 8 (0.1 μM), as measured by ELISA, using either anti-FLAG or anti-plasminogen antibody. (B) Inhibition of lysin_SM1_ binding to immobilized human fibrinogen, recombinant fibrinogen Aα variant 8, or variant 5. * ,  *P < *0.05 compared with the untreated group. (C) Relative binding of S. oralis SF100 WT or its isogenic Δ*lysin* mutant (PS1006) to immobilized fibrinogen or fibrinogen Aα variants 8 or 5. Bacteria (10^9^ CFU/ml) were incubated with immobilized fibrinogen or fibrinogen Aα variant 8 or 5 (0.1 μM). Bound bacteria were stained with 0.1% crystal violet and measured for optical density at 595 nm. Bars indicate the means (±SD) of triplicate results from a representative experiment. *,  *P < *0.05 compared with WT.

To directly compare lysin_SM1_ and plasminogen binding to the αC region, equal amounts (0.1 μM) of variants 4 (Aα_183-248_), 5 (Aα_248-376_), and 8 (Aα_534-610_) were immobilized in 96-well plates, and binding of recombinant lysin_102-198_ and purified plasminogen was measured by enzyme-linked immunosorbent assay (ELISA). As expected, lysin_SM1_ bound to variant 8, with binding reaching a plateau at 75 μM and with an apparent *K_D_* of 4.8 × 10^−5^ M (Fig. 3A). Lysin_102-198_ showed no binding activity with variants 4 and 5. In contrast, plasminogen bound to variant 5 (*K_D_* = 8.5 × 10^−5^ M), and variant 8 (*K_D_* = 1.8 × 10^−5^ M; Fig. 3A). To validate these specific interactions, we also examined whether purified fibrinogen, variant 8, or variant 5 could inhibit lysin_SM1_ binding to immobilized fibrinogen. When lysin_SM1_ (1 μg) was coincubated with 0 to 100 μM of these proteins (Fig. 3B), subsequent binding to fibrinogen by lysin_SM1_ was effectively blocked by purified fibrinogen and variant 8, but not variant 5 (Fig. 3B). In addition, we found that plasminogen (0 to 50 μM) inhibited lysin_SM1_ (1 μg) binding to immobilized fibrinogen (Fig. S3). These data indicate that the fibrinogen binding sites for both lysin_SM1_ and plasminogen are colocalized within the same domain in the αC region (Aα_534-610_) of the fibrinogen Aα chain.

10.1128/mBio.00746-21.3 FIG S3 Inhibition of lysin_SM1_ binding to fibrinogen by purified human plasminogen. Recombinant _FLAG_lysin_SM1_ was preincubated with human plasminogen (0, 0.21, 0.625, 1.85, 5.56, 16.57, and 50 μM) and transferred to 96-well plates containing immobilized fibrinogen (0.1 μM). Bound _FLAG_lysin_SM1_ was determined by ELISA with mouse anti-FLAG antibody. Download FIG S3, TIF file, 0.5 MB. Copyright © 2021 Ji et al. 2021 Ji et al. https://creativecommons.org/licenses/by/4.0/ This content is distributed under the terms of the Creative Commons Attribution 4.0 International license.

We next assessed the impact of lysin_SM1_ expression on the binding of streptococci to fibrinogen. Wild-type (WT) SF100 and its Δ*lysin* isogenic mutant (PS1006) were compared for binding to immobilized human fibrinogen and recombinant Aα truncates. As shown in Fig. 3C, the WT bound to only fibrinogen and variant 8, but not variant 5. Compared with the WT strain, PS1006 had significantly reduced binding to both fibrinogen (*P = *0.01) and variant 8 (*P < *0.001). These findings strongly suggest that lysin_SM1_ on the surface of S. oralis mediates binding to the fibrinogen αC region and that the binding domain is located within residues 534 to 610.

### Quantitative assessment of lysin_SM1_ binding to the αC region by surface plasmon resonance.

**FIG 4:**
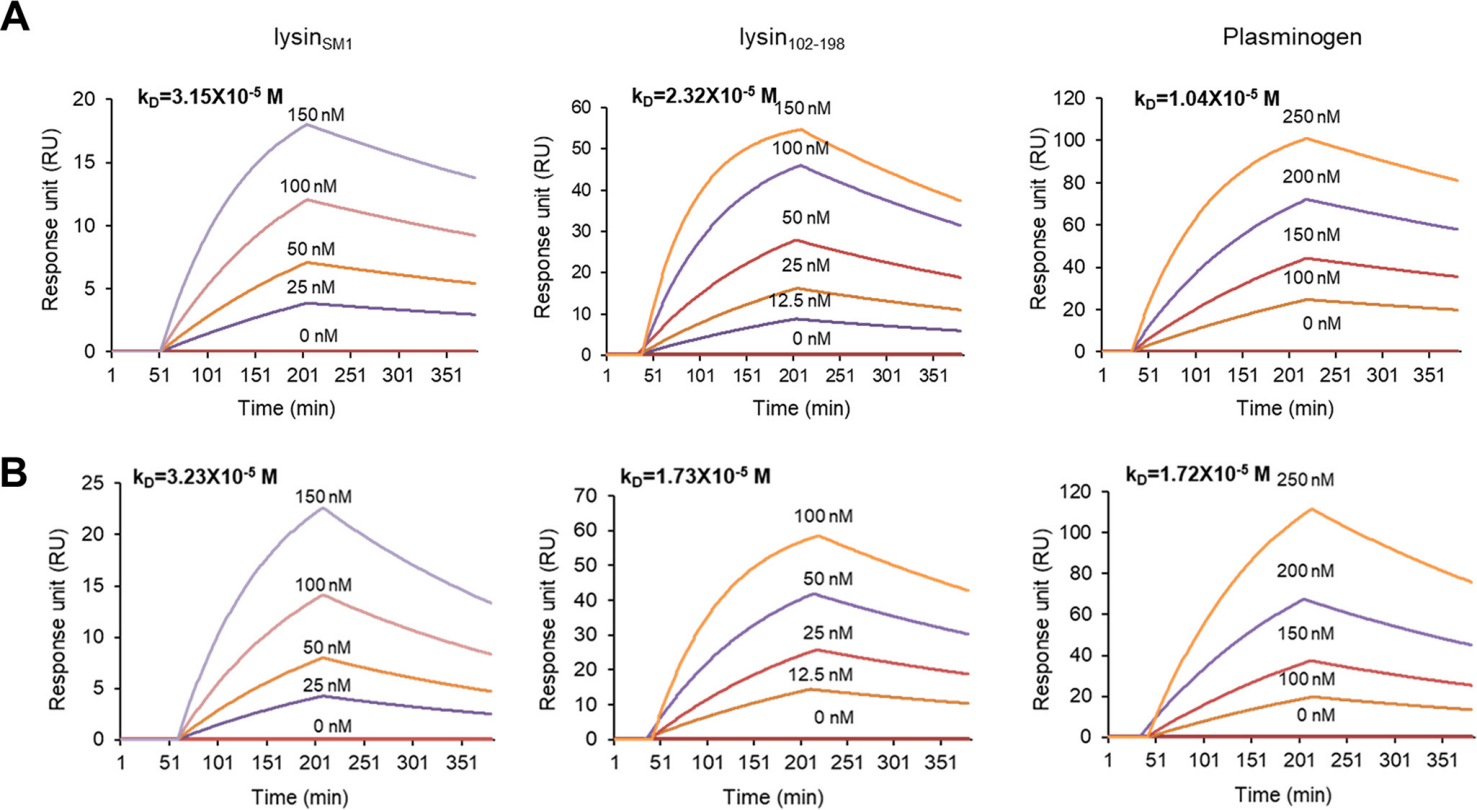
Interaction of plasminogen and lysin_102-198_ with fibrinogen and the fibrinogen αC region. (A and B) Surface plasmon resonance analysis of lysin_SM1_ (left panel), lysin_102-198_ (middle panel), and plasminogen (right panel) binding to (A) fibrinogen and (B) variant 8.

We analyzed by surface plasmon resonance (SPR) the binding affinity of lysin_SM1,_ lysin_102-198_, and plasminogen to purified human fibrinogen, by measuring the dissociation constant (*K_D_*), a specific type of equilibrium constant that measures the propensity of dissociation between two components (Fig. 4A). Increasing concentrations of lysin_SM1_ (0 to 150 nM), lysin_102-198_ (0 to 150 nM), and plasminogen (0 to 250 nM) were flowed over immobilized fibrinogen, and the *K_D_* was calculated for each protein. The *K_D_* values of lysin_SM1_, lysin_102-198_, and plasminogen to immobilized fibrinogen were determined to be 3.15 × 10^−5^, 2.32 × 10^−5^, and 1.04 × 10^−5^ M, respectively. We next analyzed the binding affinities of lysin_SM1_, lysin_102-198_, and plasminogen to recombinant forms of variant 8 (Aα_534-610_) (Fig. 4B). Lysin_SM1_, lysin_102-198_, and plasminogen showed high levels of binding to this peptide, with affinities of 3.23 × 10^−5^, 1.73 × 10^−5^, and 1.72 × 10^−5^ M, respectively. These values are within the range reported for other bacterial fibrinogen binding proteins, such as Srr1 of Streptococcus agalactiae and SdrG of Staphylococcus aureus (32, 33).

### Inhibition of plasminogen binding to fibrinogen by lysin_SM1_.

**FIG 5:**
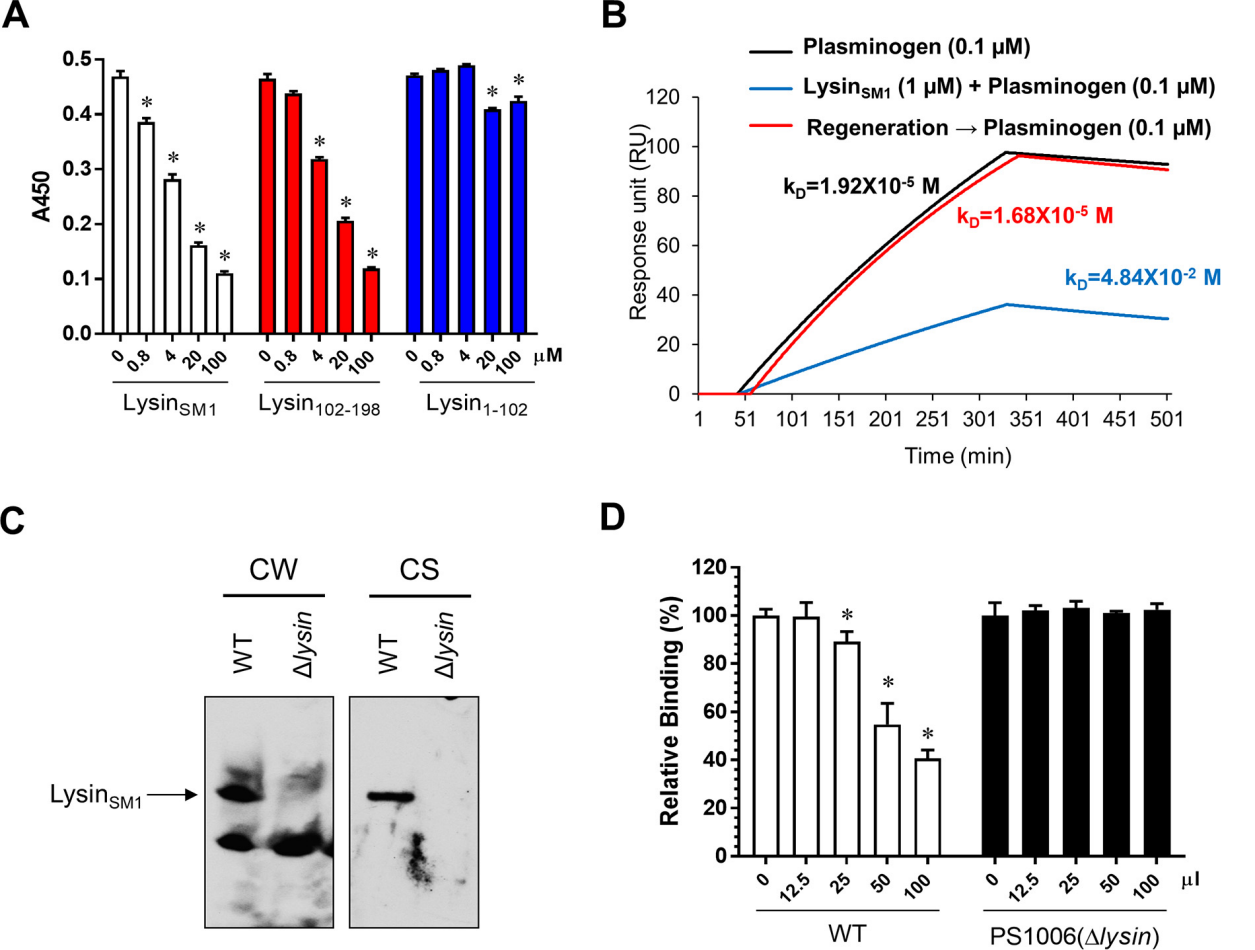
Inhibition of plasminogen binding to fibrinogen by recombinant lysin_SM1_ and secreted lysin_SM1_. (A) Recombinant lysin_SM1_ inhibits plasminogen binding to immobilized fibrinogen. Plasminogen was preincubated with recombinant lysin_SM1_, lysin_102-198,_ or lysin_1-102_ (0, 0.8, 4, 20, and 100 μM) and transferred to 96-well plates containing immobilized fibrinogen (0.1 μM). Bound plasminogen was determined by ELISA with mouse anti-plasminogen antibody. (B) Inhibition of plasminogen binding to fibrinogen by lysin_SM1_ (SPR analysis). Lysin_SM1_ (1 μM) or PBS was streamed over immobilized fibrinogen followed by plasminogen (0.1 μM). A plot of the level of binding (response units) at equilibrium against a concentration of analyte was used to determine the *K_D_*. For the regeneration studies, lysin_SM1_ bound to immobilized fibrinogen was removed by washing with glycine buffer (pH 2.0), streaming with plasminogen (100 nM). (C) Lysin_SM1_ expression in the cell wall extract (CW) or culture supernatant (CS) of S. oralis SF100 (WT) and PS1006 (SF100Δ*lysin*) was determined by rabbit anti-lysin_SM1_ IgG. (D) Inhibition of plasminogen binding to immobilized fibrinogen by lysin_SM1_ from S. oralis SF100. Wells coated with fibrinogen were preincubated with 0 to 100 μl of concentrated (10×) culture supernatant from WT or PS1006, followed by adding plasminogen (0.1 μM). Bound plasminogen was detected with antiplasminogen antibodies. Bars indicate the means (±SD) of triplicate results from a representative experiment. *, = *P < *0.05 compared with untreated group.

Since lysin_SM1_ and plasminogen bound the αC region of fibrinogen Aα with similar affinities, we next determined whether lysin_SM1_, lysin_102-198_, or lysin_1-102_ could competitively inhibit plasminogen binding to immobilized fibrinogen, as measured by ELISA. Immobilized fibrinogen was preincubated with 0 to 100 μM the lysin_SM1_ variants, followed by incubation with 100 nM plasminogen. As shown in Fig. 5A, minimal inhibition was detected for lysin_1-102_, but more than 75% inhibition of plasminogen binding to immobilized fibrinogen was seen with either lysin_SM1_ or lysin_102-198,_ which were significant compared with untreated fibrinogen (*P* < 0.05).

To confirm the above-described findings, we also examined by SPR the impact of lysin on plasminogen binding to fibrinogen (Fig. 5B). Lysin_SM1_ (1 μM) was streamed over immobilized fibrinogen followed by the addition of plasminogen (100 nM). Similar to what was seen by ELISA, the affinity (*K_D_*) of plasminogen binding to fibrinogen was 1.92 × 10^−5^ M. This was reduced to 4.84 × 10^−2^ M in the presence of lysin_SM1_. We then released the bound lysin from the immobilized fibrinogen by washing the sensor chip surface with a low-pH glycine buffer (pH 2.0). The *K_D_* value of plasminogen binding to immobilized fibrinogen on the chip surface was restored to 1.68 × 10^−5^ M, indicating that lysin_SM1_ competitively inhibited plasminogen binding to fibrinogen.

We next examined whether native lysin_SM1_ produced by strain SF100 had similar effects on plasminogen binding. As expected (21), lysin_SM1_ was found in cell wall extracts and in the culture supernatants of SF100, but not for PS1006 (Fig. 5C). We detected about 0.45 ± 0.036 μg/ml of lysin_SM1_ in the culture supernatant of SF100, as measured by ELISA. To assess the impact of lysin_SM1_ on plasminogen binding to immobilized fibrinogen, we pretreated fibrinogen-coated wells with 0 to 100 μl of supernatants collected from WT or PS1006 cultures, followed by incubation with plasminogen. As was seen with recombinant lysin_SM1_, the supernatant from WT SF100 significantly inhibited plasminogen binding (*P* < 0.006 for volumes above 12.5 μl), but supernatants from PS1006 had no effect (Fig. 5D).

### Inhibition of fibrinolysis by blocking plasminogen binding to the αC region by lysin_SM1_.

**FIG 6:**
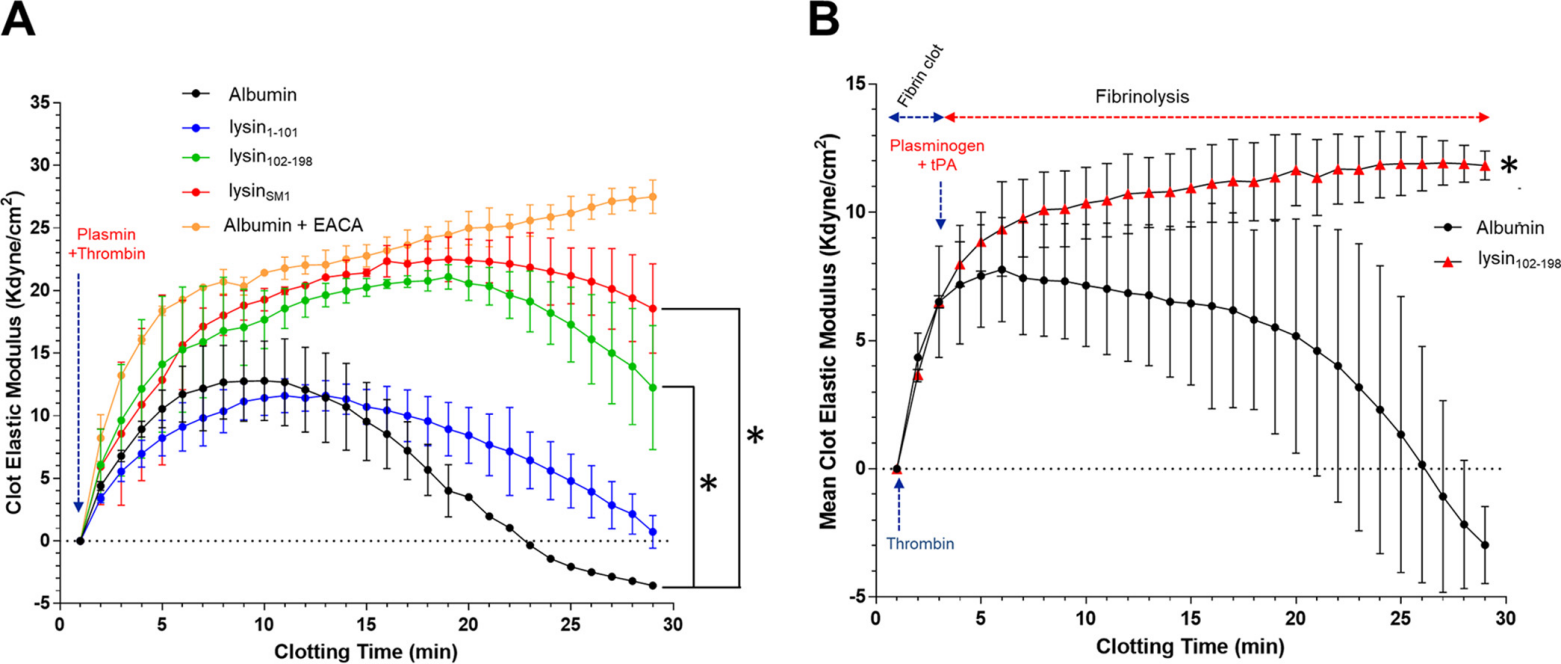
Inhibition of plasmin(ogen)-mediated fibrinolysis by recombinant lysin_SM1_. The impact of lysin_SM1_ on fibrin polymerization and proteolysis was assessed by thromboelastography (TEG). Human fibrinogen (2 mg/ml) and 13.7 μM lysin_SM1_, lysin_102-198_, or lysin_1-101_ or albumin (13.7 μM) in HEPES buffer (pH 7.4) were preincubated for 30 min at 37°C. (A and B) Each mixture was transferred to a TEG cup, followed by adding (A) thrombin (1 IU/ml) and plasmin (4 μg/ml) or (B) thrombin (1 IU/ml), tPA (0.5 μg/ml), and plasminogen (4 μg/ml). The clot elastic modulus (*n* = 2) was recorded once per minute for 30 min. *, *P* < 0.05 compared with albumin-treated group.

Fibrinolysis requires the binding of plasminogen to the C-terminal region of fibrinogen or fibrin, followed by its cleavage by tissue plasminogen activator (tPA), thereby generating the active protease plasmin (34, 35). To assess the impact of lysin_SM1_ on fibrinolysis, we examined the impact of lysin on clot formation and dissolution *in vitro*, using thromboelastography (TEG). Fibrinogen was preincubated with 13.7 μM albumin (as a control), followed by adding thrombin (to activate fibrin formation and polymerization) and plasmin (to initiate fibrinolysis). Clotting was detectable within 2 min, as indicated by an increase in the elastic modulus, and peaked at 10 min. This was followed by a decline in the modulus, indicating ongoing fibrinolysis, which reached lower than zero shear modulus strength (kdyne/cm^2^) after 24 min (Fig. 6A). Fibrinolysis was completely blocked by addition of epsilon-aminocaproic acid (EACA; 130 μg/ml), a standard lysine analogue used to competitively inhibit plasmin-induced fibrinolysis (36). When fibrinogen was preincubated with lysin_SM1_, clotting reached significantly higher levels at 10 min and peaked at 15 min. These high levels of clotting and resistance to proteolysis were sustained even at 30 min postexposure to thrombin and plasmin (*P < *0.001).

The above-described studies demonstrated that lysin_SM1_ could inhibit fibrinolysis. To determine whether this was due to the competitive inhibition of plasmin binding to the αC region, fibrinogen was preincubated with lysin_102-198_ or lysin_1-101_. When tested by TEG, preincubation with lysin_1-101_ had a minimal effect on plasmin-induced fibrinolysis. In contrast, lysin_102-198_ reduced fibrinolysis (*P < *0.001) to levels that were comparable to those seen with lysin_SM1_, indicating that the inhibition of fibrinolysis by lysin_SM1_ is due to its blocking of plasmin binding.

*In vivo*, fibrinolysis requires the conversion of plasminogen to plasmin by tPA. However, tPA can only activate plasminogen that is bound to fibrinogen. We therefore examined whether lysin_SM1_ binding to fibrinogen could inhibit fibrinolysis induced by tPA. Fibrinogen was mixed with lysin_102-198_ or 13.7 μM albumin (as a control) and incubated for 4 min with thrombin, followed by the addition of plasminogen and tPA. As expected, tPA induced extensive clot lysis when mixed with plasminogen alone (Fig. 6B). However, tPA failed to induce lysis in the presence of plasminogen and lysin_102-198_ (*P < *0.001). Since tPA can only activate plasminogen bound to fibrinogen, these data indicate that the blocking of tPA-mediated fibrinolysis by lysin_SM1_ is due to the inhibition of plasminogen binding to the fibrinogen αC region, such that tPA can no longer generate plasmin and clot lysis.

## DISCUSSION

Lysin_SM1_ is a key adhesin of S. oralis SF100, mediating bacterial binding to platelets *in vitro* through its interaction with fibrinogen on the platelet surface. However, it was unknown which regions of fibrinogen were bound by lysin_SM1_, in part because this adhesin has no structural homology to other known fibrinogen binding proteins, as measured by amino acid sequence alignment (T-Coffee) and protein structure homology-modeling (SWISS-MODEL) (37, 38). Our studies indicate that lysin_SM1_ binds residues 534 to 610 of the fibrinogen Aα chain. This differs from other known fibrinogen binding proteins of other bacteria, such as staphylococcal ClfB and Srr1 and Srr2 of Streptococcus agalactiae (both bind AA283-410 of the Aα chain), staphylococcal SdrG (AA1-25 of the β chain), and staphylococcal ClfA, FnBPA, and FnBPB (AA6-20 of the γ chain) (20, 21, 33, 39–41). Staphylococcal bone sialoprotein-binding protein (Bbp) binds the same region (AA561-575) of the Aα chain as lysin_SM1_ (42). However, lysin_SM1_ differs from at least some of these proteins in its impact on clotting. In particular, SdrG of Staphylococcus epidermidis inhibits coagulation by binding to the thrombin cleavage sites on fibrinogen (33), and Bbp of Staphylococcus aureus has anticoagulant action through an unknown mechanism via binding to AA561-575 (40). In contrast, lysin_SM1_ has no direct effect on clot formation, at least as measured by TEG, but does have strong anti-fibrinolysis effects, by inhibiting the binding of plasminogen to the αC region of fibrinogen Aα chain.

Pathogenic bacteria can produce and secrete activators or inhibitors of fibrinolysis that may impact their survival and dissemination. At least two distinct mechanisms involving plasminogen have been observed. First, plasminogen binding proteins of bacteria, such as streptokinase from group A, C, and G streptococci, staphylokinase of S. aureus, and Pla of Yersinia pestis (39–44), can bind free circulating plasminogen and convert it to plasmin (43–48). Second, proteins on the surface of bacteria, such as GAPDH (glyceraldehyde-3-phosphate dehydrogenase) and enolase of streptococci, plasminogen-binding protein (PAM) of Streptococcus pyogenes, and OspA/C of Borrelia burgdorferi, bind plasminogen, which is then converted to plasmin by tPA (49–52). Here, we report a novel mechanism for inhibiting fibrinolysis, in which lysin_SM1_ competitively inhibits plasminogen binding to the fibrinogen Aα_534-610_ region, such that it can no longer activate fibrinolysis. We also found that plasminogen bound a second region (AA248-376) within the αC region of fibrinogen. However, the binding affinity for this region was about five times lower than at the primary binding site (AA534-610). This lower affinity would explain our finding that inhibition of fibrinolysis by lysin_SM1_ did not appear to be affected by plasminogen binding to the second binding site.

Our previous studies using an animal model of infective endocarditis demonstrated that loss of lysin_SM1_ expression by SF100 was associated with decreased virulence, as measured by reduced levels of bacteria (CFU/g of tissue) within vegetations on cardiac valves, as well as in kidneys and spleens (21). Part of this reduced virulence is likely due to the loss of fibrinogen-mediated binding to platelets, which is a key step for the initial attachment of bacteria to damaged valve surfaces, as well as for the subsequent formation of infected vegetations. These structures are composed of bacteria embedded in a biofilm containing platelets, fibrinogen, and fibrin (53). Vegetation formation is thought to protect bacteria from phagocytosis and render these organisms less susceptible to antimicrobials. Moreover, larger vegetations are associated with increased embolization to target organs, such as the kidneys, spleen, and brain (54–56). Fibrinolysis may serve to mitigate vegetation formation, as both *in vitro* and *in vivo* studies have shown that tPA may reduce vegetation size and facilitate antimicrobial therapy (57–60). Thus, an additional mechanism by which lysin_SM1_ may enhance virulence is by blocking plasminogen binding to fibrinogen/fibrin within vegetations, thereby inhibiting tPA activation and vegetation lysis.

Although these studies examined a single strain of S. oralis, our findings are likely to be applicable to a broad range of organisms. Metagenomic studies by Willner et al. indicate that bacteriophage SM1 is highly prevalent in the oral microbiome and that it is the most common bacteriophage of Gram-positive organisms in the oral cavity (61). Moreover, the *lysin_SM1_* gene was among the open reading frames (ORFs) of SM1 most frequently detected. This group also examined the published salivary metagenomes from nine individuals, and all were found to contain *pblA* and *pblB*, two phage morphogenesis genes adjacent to *lysin* on the SM1 genome. More recently, metagenomic studies of salivary specimens from children detected bacteriophage SM1 in 29 of 30 individuals (62). In addition, lysin is among the most commonly expressed genes of streptococcal bacteriophages within the oral microbiome. These findings strongly indicate that SM1 or similar bacteriophages encoding a lysin_SM1_ homolog are highly prevalent in the oral microbiome (63). Our own searches for homologs of lysin_SM1_ indicate that numerous strains of not only S. oralis, but also and S. mitis and Streptococcus pneumoniae (data not shown) encode such homologs, indicating that lysin_SM1_ may be widely prevalent in a variety of streptococcal species.

**FIG 7:**
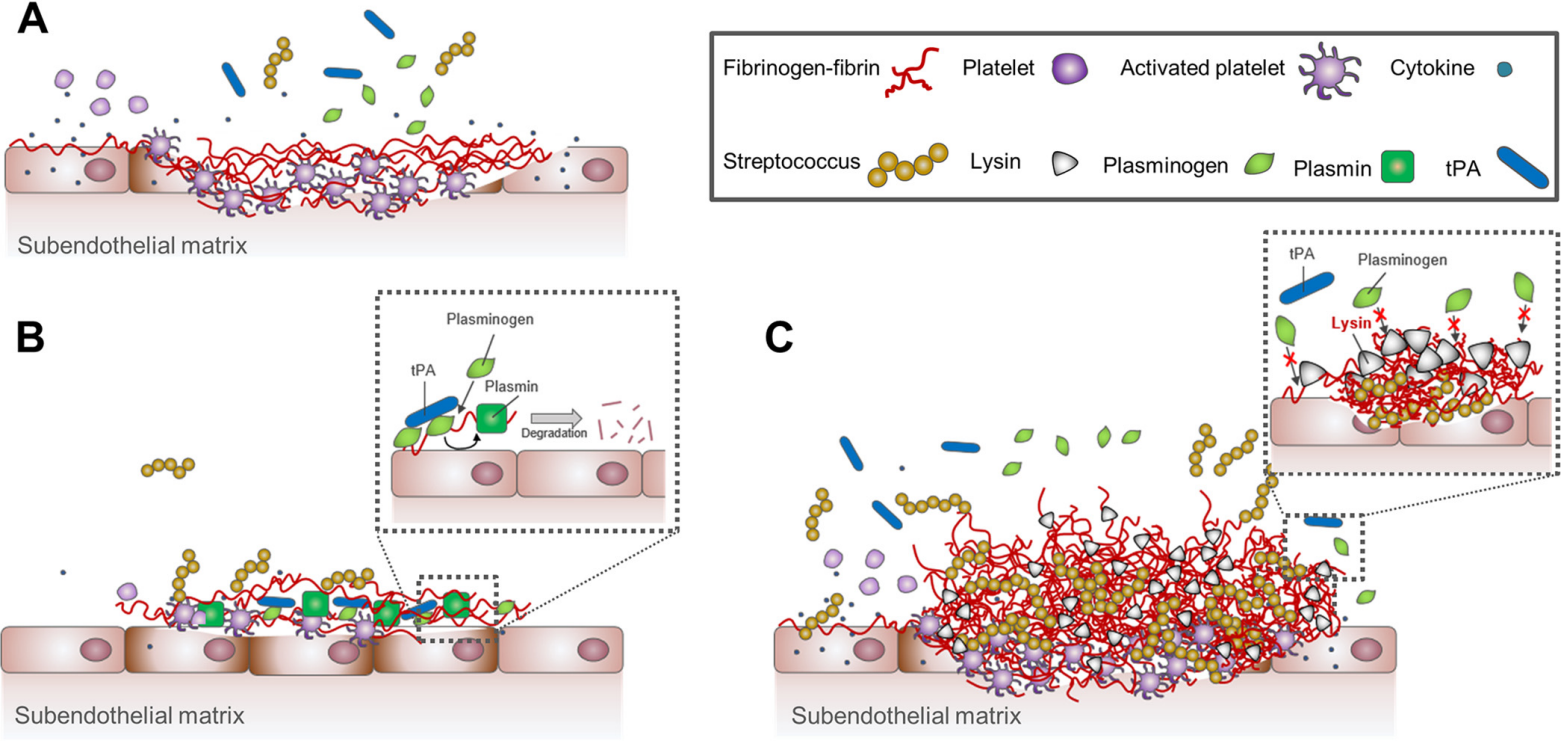
Model of the role of lysin_SM1_ during streptococcal proliferation on the surface of a damaged valve. (A) Damaged endocardium becomes covered with platelets and extracellular matrix proteins, including fibrinogen. Thrombin converts fibrinogen to fibrin polymers. (B) Streptococci circulating in the bloodstream bind to fibrin and immobilized platelets on the endovascular surface. Plasminogen binds to the αC region of the fibrinogen Aα chain, followed by recruitment of tPA, which converts plasminogen to plasmin. Plasmin degrades the fibrin clot, thereby disrupting the vegetation and releasing streptococci. (C) Exported lysin_SM1_ binds to the αC region of the fibrinogen Aα chain, thereby blocking the binding of plasminogen and inhibiting fibrinolysis.

In summary, we propose that expression of streptococcal phage lysin_SM1_ impacts the pathogenesis of infective endocarditis in the following manner: (i) damage of the endocardium by inflammation or hemodynamic trauma induces the deposition of platelets, fibrinogen, and fibrin polymerization onto the valve surface (Fig. 7A); (ii) attachment of streptococci encoding lysin_SM1_, such as S. oralis SF100, to the altered surface. This initiates endocardial infection and attachment of free or cell wall lysin_SM1_ to the αC region of fibrinogen, thereby blocking the binding of circulating plasminogen and tPA (Fig. 7B); (iii) the further deposition and polymerization of fibrinogen onto the infected endocardium along with the proliferation of bacteria on the valve surface, resulting in extensive vegetation formation (Fig. 7C).

## MATERIALS AND METHODS

### Strains and growth conditions.

The bacteria and plasmids used in this study are listed in Table S1. Strain SF100 is an endocarditis-associated clinical isolate (64). Originally identified as S. mitis by conventional clinical laboratory methods, we have recently sequenced the complete genome of this strain. BLAST analysis (v2.7.1) was carried out to identify to which species it shows similarity. The average nucleotide identity (ANI) values used to compare the genome of SF100 with S. mitis and S. oralis were determined using the OrthoANIu algorithm (https://www.ezbiocloud.net/tools/ani) (65). In addition, the whole-genome sequences were aligned with 120 bacterial marker genes, using GTDB-Tk (v1.3.0) (66), and the best-fit model was selected using ModelTest-NG (v0.1.6) (67). A phylogenetic tree was constructed for the PROTGAMMALGF model using RAxML (v8.2.12), including bootstrap analysis based on 100 replicates (68). BLAST analysis of the complete SF100 genome found the highest similarity with S. oralis ATCC 35037 (GenBank accession no. LR134336), with ANI values of 95.5% and 86.2%, respectively, for S. oralis ATCC 35037 and S. mitis NCTC12261 (CP028414). Because an ANI cutoff of 95 to 96% is used for species definition (65), these results indicated that SF100 should be classified as a strain of S. oralis. In the phylogenetic tree analysis (Fig. S4), the S. mitis and S. oralis groups were clearly separated, and SF100 clustered with S. oralis strains, further indicating that SF100 is a member of this species.

10.1128/mBio.00746-21.4 FIG S4 Phylogenetic relationships of strain SF100 of Streptococcus mitis *and* S. oralis. The numbers indicate the bootstrap value. The S. mitis and S. oralis groups were clearly distinguishable, with SF100 clustering with S. oralis strains. Download FIG S4, TIF file, 0.7 MB. Copyright © 2021 Ji et al. 2021 Ji et al. https://creativecommons.org/licenses/by/4.0/ This content is distributed under the terms of the Creative Commons Attribution 4.0 International license.

10.1128/mBio.00746-21.5 TABLE S1 Strains and plasmids. Download Table S1, TIF file, 1.6 MB. Copyright © 2021 Ji et al. 2021 Ji et al. https://creativecommons.org/licenses/by/4.0/ This content is distributed under the terms of the Creative Commons Attribution 4.0 International license.

SF100 was grown in Todd-Hewitt broth (Difco, Franklin Lakes, NJ) supplemented with 0.5% yeast extract (THY). Escherichia coli strains were grown at 37°C under aeration in Luria broth (LB, Difco). Appropriate concentrations of antibiotics were added to the medium, as required.

### Cloning and expression of fibrinogen Aα and its variants.

cDNA encoding the Aα chain of human fibrinogen was generously provided by Susan Lord (University of North Carolina at Chapel Hill) (69–71). Full-length and truncates of the Aα chain were cloned into pMAL-C2X (New England Biolabs, Ipswich, MA) with specific primer sets (Table S2) to express maltose binding protein MalE-tagged versions of variants. All recombinant proteins were purified by affinity chromatography with amylose resin according to the manufacturer’s instructions (New England Biolabs).

10.1128/mBio.00746-21.6 TABLE S2 The primers used in this study. Download Table S2, TIF file, 1.1 MB. Copyright © 2021 Ji et al. 2021 Ji et al. https://creativecommons.org/licenses/by/4.0/ This content is distributed under the terms of the Creative Commons Attribution 4.0 International license.

### Site-directed mutagenesis.

Point mutations of lysin_SM1_ were generated using a Muta-Direct site directed mutagenesis kit (Intron, Inc., Seoul, South Korea) and pET28_FLAG_-lysin_SM1_ as the template plasmid. The resulting plasmids were screened for the expected mutations by DNA sequencing (Macrogen, Inc., Seoul, South Korea). After the correct sequences were confirmed, plasmids were introduced into E. coli BL21(DE3) to express and purify recombinant forms of mutated lysin_SM1_.

### Analysis of lysin_SM1_, lysin_102-198_, or plasminogen binding to fibrinogen and its variants by far-Western blotting.

Purified human fibrinogen (Haematologic Technologies, Essex Junction, VT) and recombinant fibrinogen Aα variants were separated by electrophoresis through 3 to 8% NuPAGE Tris-acetate gels (Invitrogen, Waltham, MA) and transferred onto polyvinylidene difluoride (PVDF) membrane (Merck Millipore, Burlington, MA). The membranes were blocked with a casein-based solution (Roche, Basel, Switzerland) at room temperature (RT) for 1 h and then incubated for 1 h with FLAG-tagged lysin_SM1_, FLAG-tagged lysin_102-198_, or purified plasminogen (1 μM) in phosphate-buffered saline (PBS)-0.05% Tween 20 (PBS-T). The membranes were then washed three times for 15 min in PBS-T, and bound protein was detected with mouse anti-FLAG antibody (1:4,000; Sigma-Aldrich, St. Louis, MO) or rabbit anti-plasminogen antibody (1:3,000; Abcam, Cambridge, UK).

### Analysis of lysin_SM1_, lysin_102-198_, or plasminogen binding to fibrinogen and its variants by enzyme-linked immunosorbent assay (ELISA).

Purified fibrinogen or recombinant fibrinogen variants (0.1 μM in PBS) were immobilized overnight in 96-well microtiter plates at 4°C. The wells were blocked with 300 μl of a casein-based solution for 1 h at room temperature (72). The plates were washed three times with PBS-T, and lysin_SM1_, lysin_102-198_, or plasminogen in PBS-T was added over a range of concentrations for 1 h. The plates were incubated for 1 h at 37°C, washed with PBS-T to remove unbound protein, and incubated with mouse anti-FLAG antibodies (1:4,000) or rabbit anti-plasminogen antibodies (1:3,000) in PBS-T for 1 h at 37°C. Wells were washed and incubated with horseradish peroxidase (HRP)-conjugated rabbit anti-mouse IgG (1:5,000; Sigma-Aldrich) or goat anti-rabbit IgG (1:5,000; Sigma-Aldrich) in PBS-T for 1 h at 37°C. For some studies, wells containing immobilized fibrinogen were pretreated with lysin_SM1_, lysin_102-198_, lysin_1-102_, truncated recombinant fibrinogen variant Aα_534-610_ or Aα_248-376_, or concentrated supernatants collected from S. oralis SF100 (WT) and its isogenic mutant (PS1006 = SF100Δ*lys*) (21), followed by washing prior to the addition of FLAG-tagged lysin_SM1_. Levels of binding were assessed by absorbance at 450 nm, using 3,3′,5,5′-tetramethylbenzidine as the chromogenic substrate. Binding data were analyzed with GraphPad Prism v7.0, using a nonlinear regression curve fit and an on-site total binding equation to estimate the equilibrium binding constant (*K_D_*) for respective conditions.

### Binding of S. oralis to immobilized fibrinogen and recombinant proteins.

Overnight cultures of S. oralis SF100 and PS1006 were harvested by centrifugation and suspended in PBS (final concentration, 10^9^ CFU/ml). Purified fibrinogen or recombinant truncated fibrinogen variants (0.1 μM) were immobilized in 96-well microtiter plates and then incubated with 100 μl of bacterial suspension for 30 min at 37°C. Unbound bacteria were removed from the plates by washing with PBS, and the number of bound bacteria was determined by staining with crystal violet (0.5% vol/vol) for 1 min, as described previously (32).

### Surface plasmon resonance (SPR) spectroscopy.

SPR spectroscopy was performed using a Reichert-4 SR7500DC system (Reichert Technologies, Munich, Germany). Purified human fibrinogen (0.1 μM) in sodium acetate buffer (pH 5.5) was covalently immobilized on a plain gold surface polyethylene glycol (PEG) sensor chip. Increasing concentrations (range, 0 to 250 μM) of lysin_SM1_, lysin_102-198_, or plasminogen in PBS were flowed over fibrinogen at a rate of 30 μl/min with 3 min association and dissociation times. The sensorgram data were subtracted from the corresponding data from the reference flow cell and analyzed using Scrubber2 software (Reichert Technologies). A plot of the level of binding (response units) at equilibrium against a concentration of analyte was used to determine the *K_D_*.

### Analysis of lysin_SM1_ expression by Western blotting.

S. oralis SF100 and PS1006 were harvested by centrifugation of liquid cultures at an *A*_600_ of 0.8, and the pellet was lysed with 6 M urea. The culture supernatants were concentrated by centrifugation with Amicon Ultra-50 units (Merck Millipore). The samples were separated by SDS-PAGE with 3 to 8% Tris-acetate gels (Invitrogen) under reducing conditions and then were transferred to nitrocellulose membranes. After blocking, the membranes were incubated with rabbit anti-lysin_SM1_ IgG (1:3,000), followed by incubation with HRP conjugated goat anti-rabbit IgG (1:5,000).

### Analysis of fibrinolysis using thromboelastography (TEG).

Fibrinogen polymerization and fibrinolysis were assessed by thromboelastography, using a Hemodyne hemostasis analysis system (Haemonetics, Niles, IL) as described previously (73). This technique measures clot stiffness (“elastic modulus”) over time as an indicator of clot formation or lysis. Human fibrinogen (2 mg/ml) and 13.7 μM lysin_SM1_, lysin_102-198_, or lysin_1-101_ in HEPES buffer (pH 7.4) were preincubated for 30 min at 37°C and transferred to a TEG cup at 37°C, followed by addition of thrombin (1 IU/ml) and plasmin (4 μg/ml) or thrombin (1 IU/ml), tPA (0.5 μg/ml), and plasminogen (4 μg/ml). The clot elastic modulus (*n* = 2) was recorded once per minute for 30 min.

### Statistical analysis.

Data expressed as means ± standard deviations (SD) were compared for statistical significance using the unpaired t test with Prism v7.0 (GraphPad Software, Inc., La Jolla, CA, USA). *P* < 0.05 was considered to be statistically significant.
