# Supplementary figures and images for: Inhibition of Fibrinolysis by Streptococcal Phage LysinSM1
Source: mBio. 2021 Jun 22;12(3):e00746-21. doi: 10.1128/mBio.00746-21 (PMC8263008; doi:10.1128/mBio.00746-21)

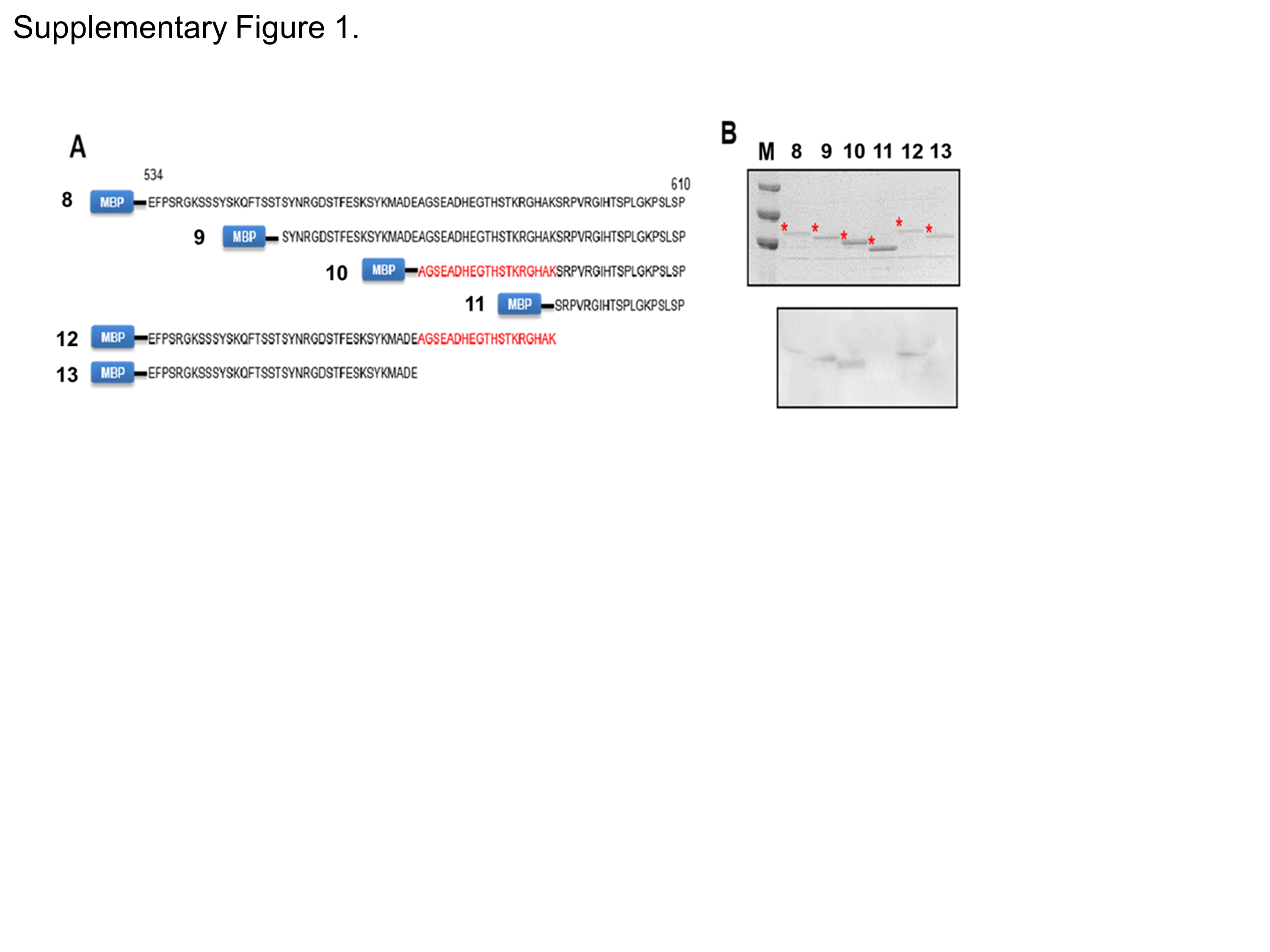

Supplement: FIG S1 [file mbio.00746-21-sf001.tif]

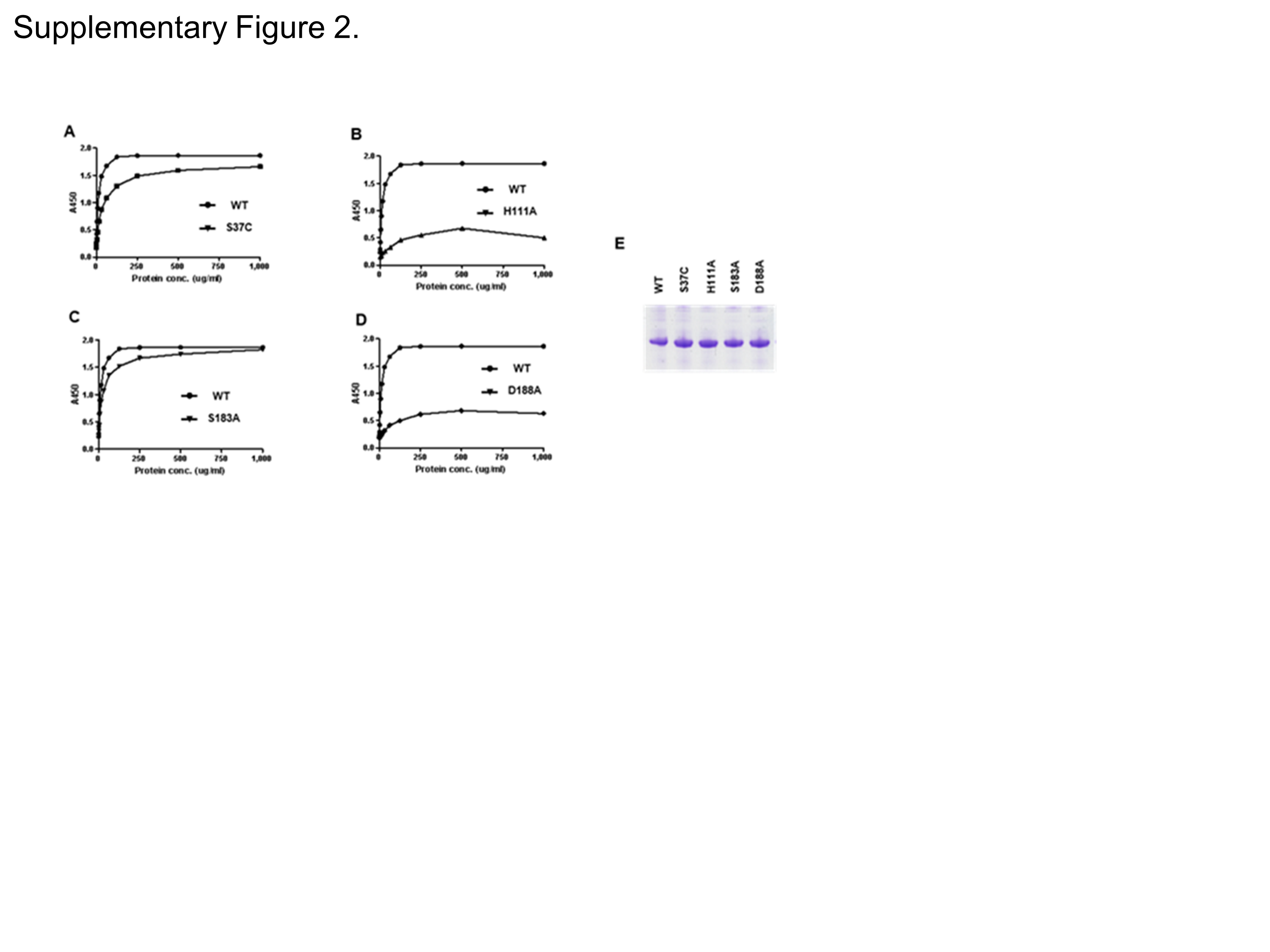

Supplement: FIG S2 [file mbio.00746-21-sf002.tif]

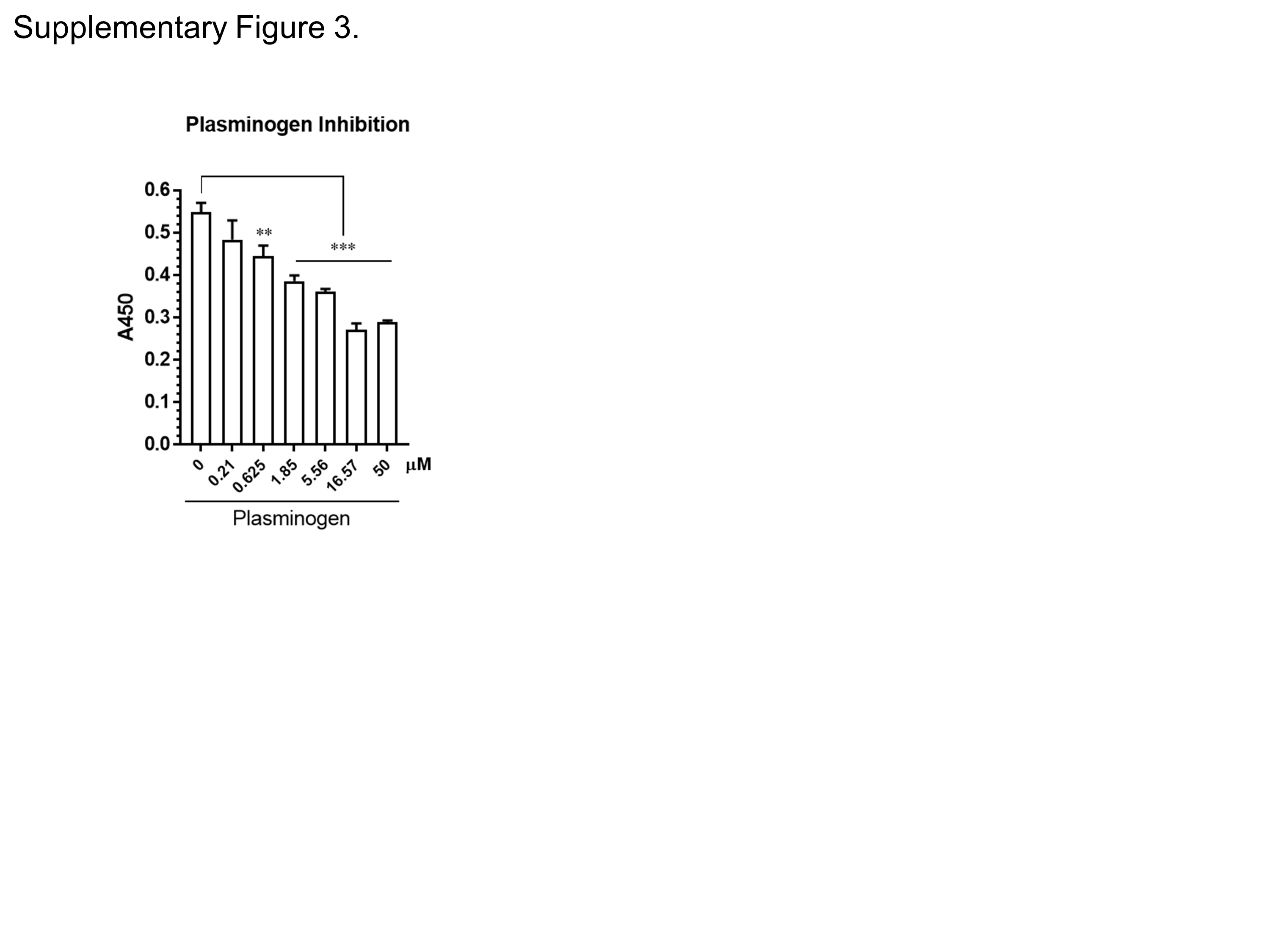

Supplement: FIG S3 [file mbio.00746-21-sf003.tif]

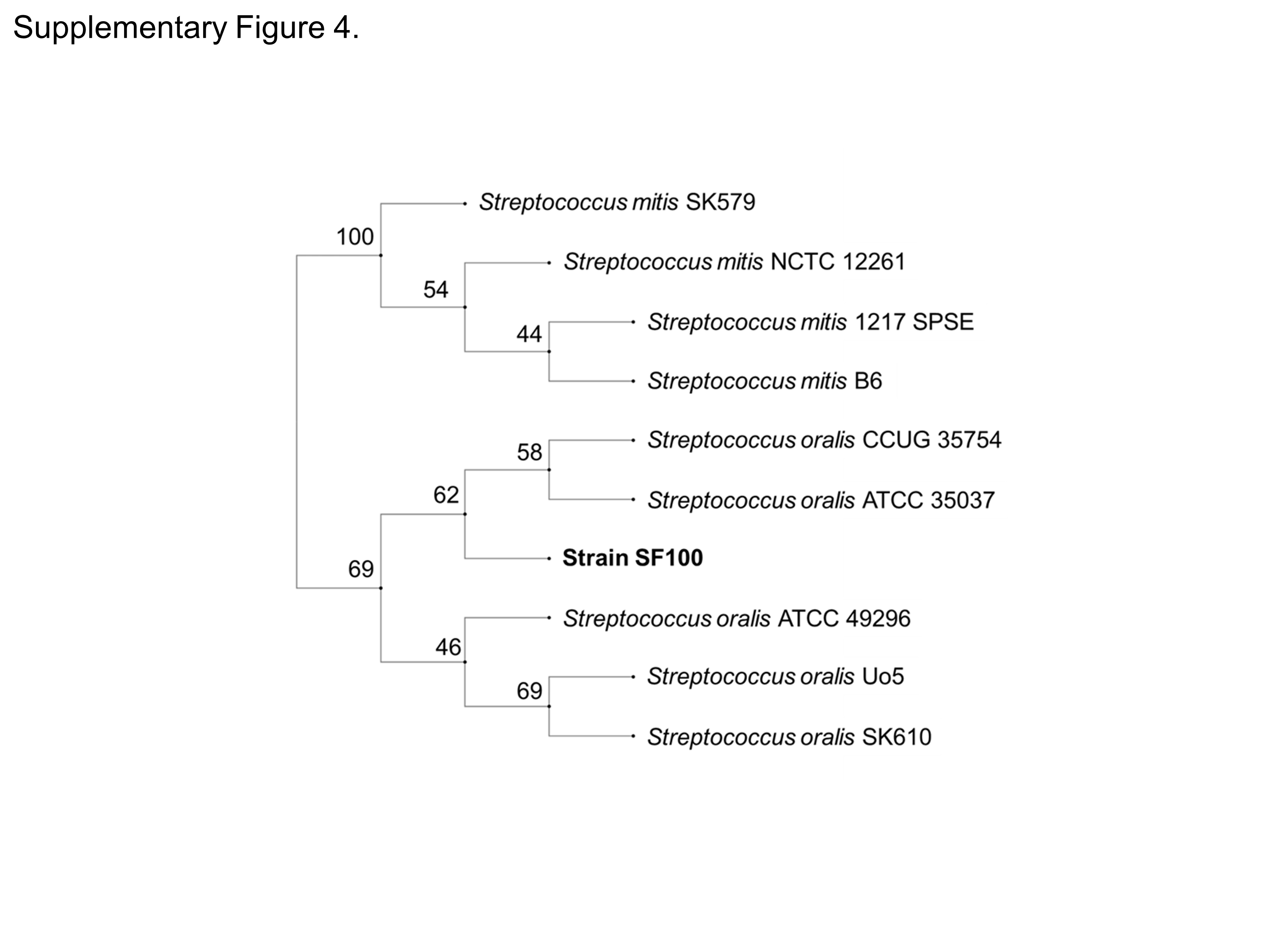

Supplement: FIG S4 [file mbio.00746-21-sf004.tif]

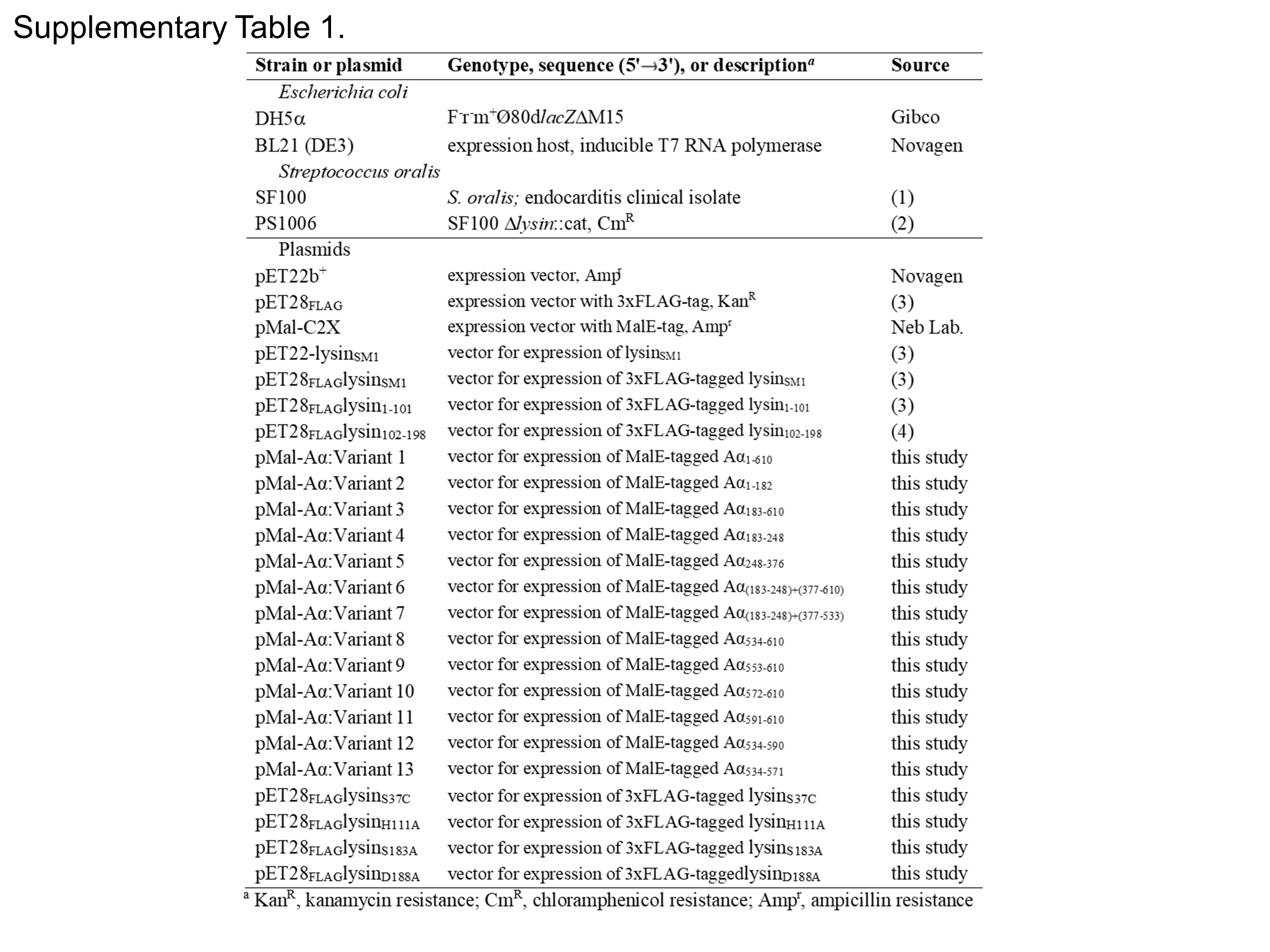

Supplement: TABLE S1 [file mbio.00746-21-st001.tif]

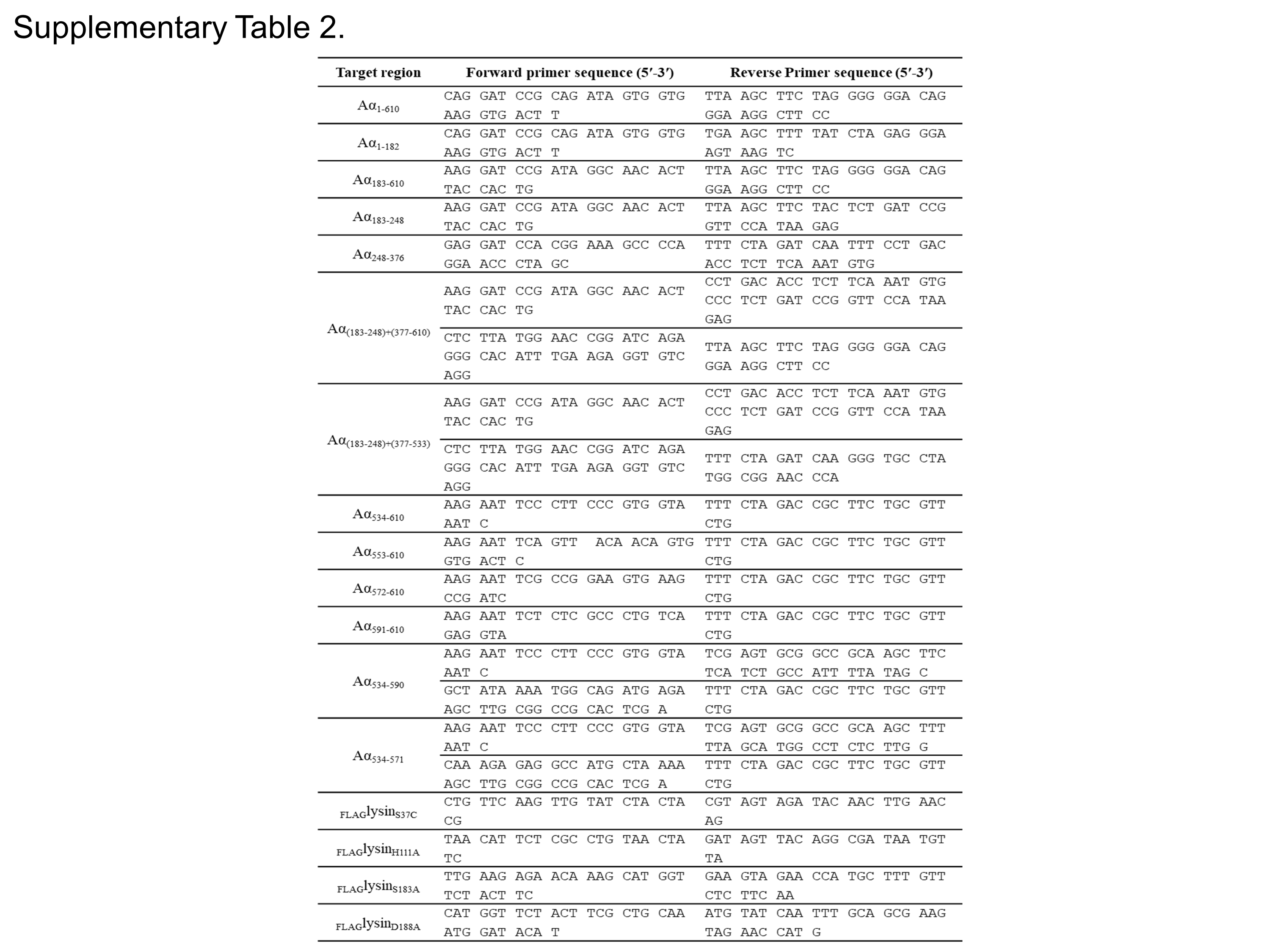

Supplement: TABLE S2 [file mbio.00746-21-st002.tif]
